# Cultural Adaptation of the Mothers and Babies Online Course for Black Mothers with Preterm Infants: A Delphi Study

**DOI:** 10.3390/ijerph22081304

**Published:** 2025-08-20

**Authors:** Kobi V. Ajayi, Robin Page, Kelly Wilson, Carly McCord, Whitney Garney

**Affiliations:** Department of Health Behavior, School of Public Health, Texas A&M University, College Station, TX 77843, USA

**Keywords:** neonatal intensive care unit, mothers, Black or African American, premature birth, cultural competency, health equity, Delphi technique, consensus development, mental health, maternal health

## Abstract

With persistent racial inequities, cultural adaptations of health programs can promote equitable maternal mental health. Despite the intersecting identities of Black mothers with preterm birth combined with racial discrimination, previous mental health programs in the neonatal intensive care unit (NICU) are void of the sociocultural context that perpetuates racially motivated care. This study uses a two-round Delphi process to gather knowledge on the cultural appropriateness and content validity of the adapted Mothers and Babies Online Course, a United States Prevention Service Task Force-recommended intervention for managing mental health symptoms in pregnant and new mothers for Black mothers with preterm birth. The Black feminist theory and cultural adaptation strategies were used to conceptualize the adaptation process. Opinions were solicited from Black mothers and professionals based on predetermined criteria. Eleven participants, comprising eight mothers and three professionals, participated in Round One. Of these, only one professional did not participate in Round Two, totaling 10 participants who participated in Round Two. The participants rated the adapted program—eMB 4 Blackmamas, positively, and four themes emerged, substantiating the importance of culturally responsive mental health. The themes also offered additional feedback related to improving the program delivery. Including diverse stakeholders in mental health research offers unique and balanced insights into designing culturally appropriate programs to promote and protect Black maternal mental health in the NICU.

## 1. Introduction

Culture is a social determinant of health, and much evidence substantiates the relationship between sociocultural factors and healthcare utilization and outcomes [1,2]. With an increasingly diverse population in the United States (U.S.), particularly among Black people [3], weaving sociocultural nuances into health programs, policies, and health communication materials can promote equitable maternal health outcomes [4,5,6].

Health inequities are seen across adverse maternal health outcomes, such as preterm birth (PTB)—births before 37 weeks of pregnancy. In 2021, Black women had about 50% higher PTB rates than white or Hispanic women [7]. In addition to adverse physical health and economic consequences associated with PTB [8,9], PTB also elicits psychological problems, regardless of racial/ethnic composition [10,11,12]. However, Black mothers experience greater mental health burdens due to racial biases compounded by intersecting identities (i.e., a combination of the PTB experiences and their racial categorization) [13,14,15,16,17]. This connotes that sociocultural determinants lead to poorer mental health among Black families in the neonatal intensive care unit (NICU). A recent study found that 73% of the 11 Black women with PTB interviewed about their mental health experience in the NICU were not offered any mental health services, including referrals [18]. Moreover, research at the intersection of PTB experiences and mental health among Black women is scarce [19].

The consequences of poor maternal mental health on mothers and infants abound [20]. Still, previous mental health prevention programs in the NICU predominantly apply a one-size-fits-all approach [21] to address psychological burdens, void of the sociocultural context wherein they thrive. Still, research on psychological interventions for racial/ethnic minority women often does not include mothers with PTB [22]. As such, the sociocultural drivers doubly impacting poor mental health issues among Black families in the NICU must be accounted for when designing public health programs to attain culturally concordant care and ultimately equitable health outcomes.

Accordingly, this study uses a two-round Delphi process to describe and present findings from the cultural adaptation of the Mothers and Babies Online course (eMB) [23] for Black mothers with PTB. Therefore, the primary objective of this study was to examine the cultural adaptations of the Mothers and Babies Online Course to enhance its relevance, effectiveness, and accessibility for Black mothers with preterm infants. The secondary objectives were to (1) identify culturally relevant modifications through expert consensus via a Delphi Study, (2) validate the adapted course with Black mothers to assess its feasibility and impact, (3) explore barriers and enablers for implementing the course in diverse settings, and (4) develop guidelines for scaling the culturally adapted course for future implementation.

### 1.1. The Mother and Babies Online Course

The eMB is an adaptation of the Mothers and Babies (MB) course [24], a United States Prevention Service Task Force-recommended intervention for managing mental health symptoms in pregnant and new mothers. The original MB is presented in a traditional face-to-face group or individual format and used in several in-person settings, including homes and hospitals. The eMB, on the other hand, is online, self-paced, and interactive. The eMB contains seven lessons and includes diverse visual content such as videos and images, vignettes, homework, and guided meditations, briefly described in Table 1. Results from a randomized controlled trial of 111 predominantly Spanish-speaking pregnant women in 23 countries showed a positive, albeit non-statistically significant, effect of the eMB in managing prenatal depression symptoms in the intervention vs. control group [25]. Both the MB and eMB are based on cognitive-behavioral and attachment theories [23,24] because they focus on the connection between thoughts and emotions, helping moms manage stress and form healthy bonds with their infants. This is particularly germane for Black mothers with a NICU experience, considering the psychological burden that accompanies PTB.

With express permission from the developer, this study utilizes the eMB owing to the flexibility and anonymity offered by online-based psychosocial interventions, which have proven effective in reducing perinatal mental health symptoms [26]. Findings from an earlier study indicated that Black mothers of preterm infants preferred self-paced online mental health programs over traditional face-to-face formats. This preference stems from the ability to fit these programs into their demanding and conflicting schedules [18]. While the MB and eMB have been adapted for several populations, including Native and Tribal American women [27,28,29], they have yet to be tailored for Black mothers or those experiencing preterm birth. The adapted eMB will be referred to as “eMB 4 Blackmamas”. This study’s findings could significantly impact mental health policies, underscoring the importance of policymakers’ role in supporting Black mothers’ mental health.

### 1.2. Theoretical Framework

This study is conceptualized through the lens of Black feminist thought [30]. The Black feminist theory recognizes the intersecting identities of Black women that expose them to racism and sexism, which in turn relegate them to the lowest hierarchy of economic, political, and social structures. It articulates several notable strategies and constructs for health researchers to situate and herald Black women’s experience in the research process to advance equitable Black health outcomes [30]. Specifically, two constructs of the Black feminist theory, voice and intersectionality, were used throughout the research process. These constructs ensured that the collective experiences of Black intellectuals, influenced by numerous sociocultural factors, were accurately represented in the development of the eMB 4 Blackmamas.

The Black feminist theory and cultural adaptation strategies employed in this study offer robust theory-informed strategies encapsulated in rigorous methodological processes to ensure the eMB 4 Blackmamas reflects the intersecting identities and distinct experiences of Black mothers with PTB. Kreuter et al. (2003) [2] as seen in Table 2. We must note that some adaptations overlapped with the cultural adaptation components.

## 2. Materials and Methods

### 2.1. Study Design

This study employed a Delphi process to solicit expert knowledge on the cultural appropriateness and content validity of the eMB 4 Blackmamas. The Delphi technique uses a consensus-based approach to gather the viewpoints of multiple stakeholders, referred to as experts with theoretical knowledge or lived experiences of a particular phenomenon in fields where the body of knowledge is still developing [31,32,33]. The Delphi process ensures experts’ anonymity and eliminates the threats of dominant and suppressive personalities in the feedback process. This technique is ideal for this study because it uses expert feedback to validate the cultural and scientific appropriateness of the adaptation, which is important for public health programs such as the eMB 4 Blackmamas.

### 2.2. Participants

We recruited participants online via social media through purposeful and snowball sampling. Participants were approached if they met the following inclusion criteria (1) possessed lived experiences (i.e., Black mothers with preterm infants and had a NICU stay in the United States) and (2) have theoretical or practice knowledge (i.e., Black professionals who have worked with the mothers actively in the past 12 months in different capacities—researchers, healthcare providers, or advocacy/policy efforts). The terminologies “experts” and “participants” are used interchangeably throughout this study, implying mothers and professionals alike. Using diverse perspectives offers holistic and balanced viewpoints of the individual and system-level factors contributing to mental health problems among the population under study. Given that there is no established consensus on the sample size for Delphi methodologies [32,33], our sampling strategy was ideal for this study.

### 2.3. The Adaptation Process

As outlined in Table 2, we combined different “strategies intended to reach one specific person [Black mothers with preterm infants], based on characteristics that are unique to that person [them], related to the outcome of interest [mental health], and have been derived from an individual assessment” [2]. Specifically, we designed the eMB 4 Blackmamas iteratively by (1) reading the eMB in its entirety and deliberating internally to understand the extent to which adaptation should be made to ensure we do not deviate from the validity of the content itself, (2) identifying the aspects of the eMB that require changes, (3) changing and creating new materials, and (4) conducting external consultations to ensure validity and reliability. In line with Black feminist thought, we referred to the results from our earlier study, where we specifically asked mothers to recommend strategies for developing a culturally appropriate mental health program for the NICU [18]. To avoid deviating from the core scientific validity of the eMB, we retained the flow, lesson titles, and core foundational concepts (i.e., the three main components of cognitive-behavioral therapy (CBT)—pleasant activities, thoughts, and contact with others—attachment theory, and mindfulness practices) of the eMB 4 Blackmamas.

### 2.4. Data Collection and Delphi Rounds Procedure

This Delphi study employed a two-round iterative process and was hosted on Qualtrics XM. Participants were invited to participate via targeted social network sites (e.g., LinkedIn and several Facebook groups for Black people) using snowball and convenience sampling. An initial sign-up form containing the inclusion and exclusion criteria and background information about the study was sent to individuals via email who showed interest in the study. The form was used as a proxy to screen participants. Individuals who met the study criteria and agreed to participate in all rounds were emailed with additional information about the study, including the informed consent document and an anonymous hyperlink to the online Delphi questionnaire. Incentives were provided on a graduated basis: USD 10 for completing Round One and USD 30 for completing Round Two, totaling USD 40 for both Rounds. Experts were allowed to choose between an Amazon or a Target gift card.

### 2.5. Delphi Process Round One

Round One contained sociodemographic questions, five-point Likert scale questions (strongly agree, somewhat agree, neither agree nor disagree, somewhat disagree, and strongly disagree), a rating scale (0–100; higher scores represent positive values), and open-ended questions. Participants responded to questions using the Likert scale to describe (1) if the updated images and storyline of the materials (e.g., vignette comprising contrasting scenarios portraying positive vs. negative adaptations for Black mothers in the NICU feeling stressed, anxious, or depressed) were useful, appropriate, easy to understand, and relatable in the NICU context; (2) if the updated video verbiage (a) represented the experiences of Black mothers, (b) represented the NICU experiences, (c) were easy to understand; and (3) if the additional resources and textual contents were important, helpful, useful, relevant and represented the experiences of Black mothers and the NICU hospitalization.

Participants were also asked to rate their preferred presentation format of the videos on a scale of 0–100 (e.g., an actual person, a slide with images, or combined). Using the open-ended questions, participants then provided feedback on each lesson. At the end of the survey, they were asked to raise comments, suggestions, or other concerns about the program. The Round One questionnaire was pilot-tested for face validity by graduate research assistants and one mother, who met the study criteria voluntarily. An example of some of the questions asked in Round One is included in the Supplementary File. The information gathered in Round One was used to improve the eMB 4 Blackmamas and to develop the questionnaire distributed in Round Two of the Delphi process. A document containing the anonymized summary of the expert’s responses was also created and included for review in Round Two.

### 2.6. Delphi Process Round Two

Round Two contained only questions from Round One, where the experts had divergent views, requested changes, or new information to the initial adaptation. As a result, questions that reached consensus in Round One were not included in Round Two. Additionally, in Round Two, we aimed to achieve a final consensus and resolve divergent viewpoints on statements where an agreement had not been reached. The questionnaire contained Yes or No questions and open-ended questions only. We used tracked changes (e.g., redline) of statements and updated images, as well as the materials from Round One, to make experts aware of the specific revisions.

### 2.7. Data Analysis

A two-process analysis approach was used. First, we analyzed the sociodemographic characteristics of the experts on Excel using descriptive statistics. Using counts and percentages, we grouped and analyzed the data separately by expert traits (i.e., mothers vs. practitioners) to quantify and compare the Likert scale ratings and statements and to investigate any divergence in the responses. Following previous studies [34], we defined consensus as 70% or more agreement on the two top measures (strongly agree and somewhat agree) on the Likert Scale; these were not included in the subsequent round. On the other hand, responses of more than 70% on the last two measures (somewhat disagree and strongly disagree) were returned for rating in Round Two.

Next, we conducted an inductive thematic analysis of the open-ended responses to provide additional insights into the feedback provided. At first, the data was read entirely to get a sense of the salient data points. An iterative process involving reading the data to identify patterns within the data was used to develop codes, themes, and sub-themes. One researcher then manually coded the data using the coding book approved by the team. We triaged the Likert scale responses with the open-ended responses; comments with a negative undertone, confusion, or requesting clarifications, regardless of whether they had reached consensus, were revised by the team and returned for re-rating, depending on the extent of revision done.

## 3. Results

We invited 17 experts (12 mothers and 5 professionals) to participate in this study. Of these, 11 (65%) participated in Round One, comprising 8 (72%) mothers and 3 (27%) professionals (Table 3). Mothers’ ages ranged between 22 and 43 years; were married/living with a partner (6/8; 75%); had their baby through emergency cesarean (6/8; 75%); had an annual household income of >USD 70,000 or more (5/8; 63%); and had a graduate degree or more (5/8; 63%). The longest time spent in the NICU was 109 days, and the birth ranged between 29 and 35 weeks of gestation. Among the professionals, all were females (3/3; 100%), had a graduate degree or more (3/3; 100%), and worked as mental health professionals (2/3; 67%) and in an advocacy organization (1/3; 33%).

### 3.1. Delphi Round One

In Round One (*n* = 11), experts generally endorsed the adaptations made to the eMB 4 Blackmamas, with most of the responses in the strongly/somewhat agree (330/374) vs. neither agree nor disagree (35/374) and strongly/somewhat disagree (9/374) category of the survey items. Positive responses were related to the usefulness, understandability, relevance, and appropriateness of the eMB 4 Blackmamas contents. This feedback was reflected in the open-ended comments where participants, particularly mothers, expressed experiencing similar scenarios highlighted in the materials. However, in some cases, there were conflicting views between mothers and providers, with the providers asserting that they had never seen the scenarios highlighted in their practices. For example, concerning the Djenne and Kenya Days story, one mother noted that “I [she] felt very similar about my [her] NICU experience. Similarly, a provider mentioned that “Those days [Djenne and Kenya Days] are possible; more often than not. The struggle I see as a practitioner is that they don’t know who to call due to lack of support for other issues in the past and don’t want to bear judgment.”

Concerning the presentation format, participants responded favorably to having an actual person they can see present the videos (overall mean score = 88.0) over a slide with only the presenter’s voice (overall mean score = 38.9). Even though participants preferred the use of an actual person, they were split on who should present the videos, with some endorsing only mothers and others a combination of mothers and mental health providers. For example, one participant, a mother, said,

“*I think a Black mother with preterm birth should present the video. Sometimes, health professionals cannot relate to us at the level we need. We often feel unheard and judged by providers. It is easier to empathize with someone who has a like background. were supported by the feedback received.*”

Whereas a provider noted

“*I think a combination of professional and Black mothers experienced with preterm birth would be helpful as presenters. It would bring a good balance of relatability and expertise.*”

Of the 34 Likert-scale questions asked in Round One, three items did not achieve consensus, with less than 70% agreement scores on the strongly/somewhat agree category, and were triaged for substantial edits for Round Two. However, we also edited items with negative feedback and important suggestions for improvement and returned for re-rating in Round Two.

### 3.2. Delphi Round Two

A total of 10 (10/11, 91%) participants, comprising eight mothers and two professionals, completed Round Two of the Delphi study. Round Two had fewer questions (*n* = 11) containing new and edited items of the eMB 4 Blackmamas. The modifications to Round Two were related to clarity in the Djenne and Kenya Days storyline, terminologies, additional vocabulary to improve the context of the eMB 4 Blackmamas, and the decision on who, between mothers or healthcare professionals, should present the videos. Round Two had several yes/no answer choices, one open-ended question to solicit participants’ final feedback, and preferred choice questions from two alternatives. However, we included a set of Likert scale questions due to the skip logic omission of one of the questions from Round One.

Concerning the video presentation, seven (70%) participants endorsed combining mothers and health professionals and switching the presenters based on the content and storyline of the program, vs. three (30%) who approved only combining mothers and health professionals in all the videos. All the participants endorsed all the questions asking for participants’ preferred choices from the alternative text and scenarios. For example, in Round One, participants noted that the adaptations might not connect with families who do not have family support. As a result, we included verbiage in Round Two to acknowledge this limitation. We revised the statement to empathize with mothers without family support while recommending alternative options such as virtual support or community-based organizations (see Table 4). Participants generally strongly/somewhat agreed (28/29) vs. one participant who somewhat disagreed (1/29) with the additional Likert scale question.

A consensus was achieved with scores greater than 70% agreement in the strongly/somewhat agree category. Furthermore, participants’ feedback was favorable as one participant noted: “*I think this will be a great resource for mothers with children in the NICU. I wished there was something similar available when my children were there.*”

### 3.3. Thematic Analysis

Four major themes were inductively generated based on the open-ended comments using thematic analysis.

*Theme One: Dichotomy Between Professionals and Mothers’ Perspectives*

We found that professionals often thought some adaptations might be unrealistic or extreme. However, mothers mainly perceived that the experiences captured throughout the eMB 4 Blackmamas accurately reflected their experiences. For instance, in response to one of the Djenne and Kenya Days about partner communication and support, a professional said the following:

“*From professional experience, I haven’t heard any of these narratives.*”

However, a mother said

“*I can relate to both of them.*”

*Theme Two: Diverse Needs and Multiple Experiences in the NICU*

Participants, professionals, and mothers alike reiterated that while the eMB 4 Blackmamas addresses a critical aspect of the NICU experience, mothers have diverse needs and experiences, which might be challenging to manage through the eMB 4 Blackmamas without deflecting from the core contents of the program. One mother said

“*I think there is more that should be discussed in this section. I struggled to pump milk early on and felt like I was failing because it was my only job. I learned how stress and eating (or not eating) affected my ability to pump. You must remember to advocate for your baby, get proper sleep, find support, and go home and rest to be your best self.*”

One participant describes this dilemma succinctly:

“*It’s hard to say as the experience is going to vary so much from person to location to other factors in their lives.*”

Yet, another said, “*It could be helpful to include some perspective about thoughts of racism in your child’s care causing anxious thoughts as well.*”

*Theme Three: Need for an Inclusive NICU Care Program*

Participants reiterated the importance of creating an inclusive NICU care programing that incorporates partners’ needs and the needs of other support networks. For example, one participant buttressed the addressing partner support. They mentioned that “*it can be a difficult topic to address because there are so many types of partners, as well as those without partners.*”

Nonetheless, all the participants praised the inclusion of same-sex couples in the narratives, as highlighted by some participants who said,

“*I appreciate the inclusion of diverse families” and “Being inclusive of same-sex couples is great.*”

*Theme Four: Going Beyond Individual to Structural-level Influence*

While participants “*believed that the eMB 4 Blackmamas is very culturally appropriate*” and that the “*storylines have great real-life implications,*” they stressed the significance of promoting languages, sociocultural bias, and other idiosyncrasies that reflect Black families in the narratives. Some of these include the inclusion of medical mistrust in the Black community, representation of same-sex vs. heterosexual parents, using empathetic language, and acknowledging difficulties young Black mothers encounter getting validation from older Black women because older Black women cannot provide “*sensitive hand holding or support*” due to their upbringing. However, in general, participants believed that the “*resources were very informative and would be helpful to mothers of preterm children because some of the tips mentioned were things mothers dont think of because they are often focused on the health of their children.*”

## 4. Discussion

A two-round Delphi study was conducted to validate the cultural appropriateness of the original eMB course for Black mothers with PTB and to explore the level of consensus among experts. Similar to the literature, our study found that incorporating social and cultural determinants of health in maternal health research and programs, undergirded by the viewpoints of mothers and diverse stakeholders (not just health providers), offers unique and balanced insights into designing culturally appropriate programs to promote and protect Black maternal health [34,35]. Moreover, to the best of our knowledge, this study is the first to create a mental health program specifically for Black mothers in the NICU, which is crucial given the intersecting identities of Black women, perpetuating poorer mental health and maternal health outcomes.

Digital health interventions are ubiquitous in today’s technology-driven climate, even more so among individuals struggling with mental health problems [36]. However, with its presence comes concerns about the digital divide in healthcare access because of limitations of patient representation in designing and implementing digital health solutions [37]. As a result, it is essential to design psychosocial digital health interventions that are culturally appropriate to enhance usability and overall clinical effectiveness, particularly to increase access to care for Black mothers [38]. Singular cultural adaptation approach (i.e., changes to languages only) of health programs and interventions may not address the complex health needs of minority populations. Instead, combining several cultural adaptation strategies, including the use of Delphi methodologies, can potentially improve Black maternal health while also promoting inclusivity, program acceptance, and efficacy. Moreover, Black mothers with PTB prefer self-paced digital mental health programs to traditional face-to-face programs because such programs fit into their conflicting and demanding schedules [18].

### 4.1. Main Findings and Future Research

The thematic analysis found several interesting themes with implications for health disparity research and programming. Even though our sample included participants from the same ethnic groups, perceptions between the mothers and professionals were divergent. This finding corroborates the literature [34] and is unsurprising when considering that professionals’ opinions are often influenced by textbook training and the client’s experiences. And given that they only spend a short amount of time with mothers, primarily during medical consultation (e.g., during counseling, rotations) or advocacy events, more than likely, their knowledge about how mothers experience stress in and outside of the NICU is limited. This by no means undermines the importance of including professionals or individuals who share similar social characteristics in public health programs. Still, it is a caveat for future researchers and program planners to consider when designing public health programs or policies. As such, beyond including individuals from a particular race/ethnicity, researchers and program planners must ask pertinent questions, such as what is the composition (e.g., socioeconomic status, years of experience, field of expertise, or rural vs. urban region) of professionals included in this research/program and how might they accurately represent the voices of mothers?

Like past research [19], this study found that the needs of Black families in the NICU are diverse and influenced by numerous distal and proximal factors that are internal and external to the mother’s locus of control, with mental health consequences. Thus, the eMB 4 Blackmamas may not be able to address all the needs of Black mothers in the NICU. Instead, a collaborative and multidisciplinary approach that incorporates different health services with community partnerships may be appropriate. While this approach has been successful in traditional offline settings, such as the National Network of Perinatal Quality Collaboratives, an initiative of the National Institute for Children’s Health Quality [39], there is room for innovative digital health strategies that can link users based on their health needs to different health services, referrals, or community organizations from one platform. This is important because NICU families often interact with multiple providers. Moreover, when considering Black families, it is pertinent to incorporate mediums beyond the traditional medical care model of holistic health that appeal to their sociocultural needs. Such services may include faith-based services or non-traditional health practices.

Another noteworthy finding is the significance of incorporating sexual minority individuals in mental health programs in the NICU and the obstetric setting. Although sexual and gender minority populations have elevated levels of mental health problems compared to their counterparts, the prevalence is higher among racial and ethnic minorities. Yet, inclusive mental health interventions remain scant, particularly for obstetric patients [40,41]. Thus, as reiterated by some participants in the study, the inclusion of same-sex couples in the eMB 4 Blackmamas is ideal and remarkable because such programs are fundamental to addressing escalating mental health burdens among this population. Therefore, future public health programs and interventions in the NICU should consider designing programs through a gender-inclusive using an expansive lens, regardless of the targeted population.

Persistent medical neglect and disenfranchisement of Black women are substantiated using the Black feminist theory to elucidate the connection between race/ethnicity and health. Moreover, the interplay between culture and health, particularly regarding mental health among Black populations, warrants that public health research and programs are undergirded by methodological approaches, such as the Delphi technique, that affirm their distinct experiences. For instance, the historical and contemporary racial discrimination towards Black women has resulted in medical distrust and apathy to seek and utilize psychotherapy [42]. Further, discriminatory healthcare practices and societal and mainstream media stereotyping of the “Angry Black woman” are linked with misrepresentations and mischaracterizations of Black women [42]. This negative stereotyping subjugates and constrains Black women from expressing negative feelings or any character in similitude to the Angry Black woman phenomenon, which in itself is a recipe for mental health illnesses. It is, therefore, not far-fetched to find Black mothers with NICU experience refusing to disclose mental health symptoms to clinicians who may not be culturally trained, as inferred by mothers in this study.

### 4.2. Strengths and Weaknesses

This study expands the literature and advocates for culturally competent mental health care in the NICU. Using a Delphi approach with perspectives from mothers and professionals strengthens our work. It brings new perspectives into the needs of Black mothers with PTB while offering a blueprint for future mental health programming in the NICU, with implications in the broader obstetric environment. Another unique strength is combining multiple approaches to culturally adapt the eMB 4 Blackmamas. However, this study is not without limitations. Even though this study provides a balanced view based on participant characteristics, the generalizability of the results might be limited due to the sample size, combined with the small number of professionals with a limited area of expertise and years of experience. Another potential limitation of this study is that participants were recruited from the Internet and high socioeconomic strata. As a result, the feedback received may have differed if the participants were of low socioeconomic status. However, given that our findings corroborate the literature, we do not see this as likely to impact the feedback received. Similar to other online psychological interventions, certain populations, particularly those without access to internet-enabled devices or unstable internet networks, are systematically excluded from participating in such programs. We also did not include support systems (e.g., family members) in our panel of experts, which may have offered additional insights into the eMB 4 Blackmamas, considering that the program encourages the participation of partners or families. In addition, this study was carried out solely online, limiting our ability to promote expert discussions on divergent views. However, we attempted to mitigate this limitation by summarizing the feedback (divergent and consensus) from Rounds One and Two of the survey to the experts and encouraging comments. Further, while our study is the first to culturally design a mental health program for Black mothers with preterm infants, the program has yet to undergo feasibility or pilot testing to evaluate its efficacy in managing depressive symptoms. As a result, we may not fully understand the barriers to implementing the course, whether in clinical or community settings.

### 4.3. Public Health Implications

This study used a two-round Delphi methodology with mothers and professionals to adapt the Mothers and Babies Online course culturally. Our findings have implications for patients, health systems, program planners, and health policy. Culturally appropriate web-based mental health programs, such as the eMB 4 Blackmamas, in the NICU setting can empower Black mothers to take charge of their mental health. For instance, seeing other mothers who look like them discuss their NICU experiences can make them feel seen and their concerns validated. The eMB 4 Blackmamas increases awareness about perinatal mental health while contextualizing the sociocultural environment that leads to elevated mental health problems that are often not addressed in the hospital setting.

The program also has implications for closing gaps in mental health treatments and other barriers for Black families. Providers could incorporate the eMB 4 Blackmamas as referral sources for mothers in the NICU. It could also be used as a group intervention to facilitate peer support and to build community in the NICU. The program could also be embedded into hospital electronic medical records for easy accessibility. Community partners can also use the program alone or alongside other health education programs (e.g., breastfeeding education curriculum) in their practice. While this program focuses on a particular racial/ethnic group, the eMB 4 Blackmamas can serve as a template for future adaptations for minority populations.

The eMB 4 Blackmamas demonstrates the imperative for culturally concordant public health promotion activities. Policymakers and program planners must also consciously promote equitable health communication materials when designing health interventions to encourage dose program response, effectiveness, and equitable health outcomes. The eMB 4 Blackmamas offers a blueprint for creating culturally appropriate public health programs to advance Black maternal health.

## 5. Conclusions

This study demonstrates the importance of culturally adapting psychosocial interventions to meet the unique needs of Black mothers with PTB. These needs include centering care with the professionals they can identify with, flexibility to accommodate demanding schedules, culturally relevant content that reflects lived experiences, and strategies to address stigma around mental health care in the NICU. By tailoring the Mothers and Babies Online Course, the research emphasizes improving accessibility, relevance, and maternal mental health outcomes. The Delphi technique ensured that expert consensus informed these adaptations, making the course inclusive and impactful.

## Figures and Tables

**Table 1:** Table showing a brief overview of the Mothers and Babies Online Course [24].

Lesson	Lesson Title	Description	Overview of Each Lesson
#1	Purpose and Overview	This lesson introduces the guiding principles of the Mothers and Babies Online Course (eMB).	Video using human images and/or other visual materials introducing the eMB, discussing stressors that affect the mother and baby relationship, and the goals of the program. Quick Mood Scale. Information on the sources of stress in the perinatal period. Violet and Mary Days. Personal project.
#2	Thoughts and My Mood	This lesson discusses the implications of different types of thoughts, how to identify helpful and harmful thoughts, and strategies for developing helpful thoughts.	Video using human images and/or other visual materials discussing personal reality—how thoughts and moods affect inner reality. Quick Mood Scale. Violet and Mary Days. Types of thoughts. Interactive exercises, referral to additional resources in the eMB, and personal project.
#3	Fighting Thoughts	This lesson discusses how thoughts impact moods and how to reduce harmful thoughts.	Video using human images and/or other visual materials discussing fighting harmful thoughts and moods and how to develop helpful thoughts. Quick Mood Scale. Tailored message for pregnant and postpartum women. Interactive exercises, referral to additional resources in the eMB, and personal projects.
#4	Activities and My Mood	This lesson teaches participants how to identify and develop pleasant activities to improve their moods.	Video using human images and/or other visual materials discussing pleasant activities and how they affect personal reality—external reality. Quick Mood Scale. What I want to do vs. what I have to do. Interactive exercises, mindfulness practices, referral to additional resources in the eMB, and personal projects.
#5	Pleasant Activities	This lesson teaches participants to engage in pleasant activities with their babies to improve their moods. It also teaches participants about babies’ developmental milestones and helps them learn about age-appropriate activities.	Quick Mood Scale. Textual content about how babies learn, how mothers can engage in pleasant activities for a healthy mother-baby relationship, and how to overcome challenges. Interactive exercises, referral to additional resources in the eMB, and personal projects.
#6	Contact with Others and My Mood	This lesson continues to discuss pleasant activities by focusing on developing healthy relationships with others. It teaches communication styles, how to identify different support systems, and how our communication style can affect moods and relationships. It also describes how mood affects interaction with others and vice versa.	Video using human images and/or visual materials discussing how contact with others, including social networks, can affect moods. Quick Mood Scale. Violet and Mary Days. People in My Life and the Ways They Support Me. Communication Style and Mood. Interactive exercises, referral to additional resources in the eMB, and personal projects.
#7	Graduation	This lesson reflects on the course and planning for the future and ends with a graduation certificate.	Discussion about planning for the future and graduation. Violet and Mary Days. Interactive exercises, referral to additional resources in the eMB, personal project, and certificate.
	Additional Resources	Catalog of relaxation and meditation exercises.	

**Table 2:** Strategies and examples of the cultural adaptations to the eMB 4 Blackmamas.

Cultural Adaptation Strategies	Description of the Strategies	Changes Made to eMB 4 Blackmamas
Peripheral	Peripheral adaptations give programs or materials the appearance of cultural appropriateness by making them appeal to a given group. This may include using certain colors, images, fonts, pictures of group members, or declarative titles.	Examples of peripheral adaptations made to the eMB included the following: Changes to the name of the course to reflect the population “eMB 4 Blackmamas.” Used pictures of Black people throughout the eMB and in all accompanying materials. Changes from Violet and Mary Days (a vignette of two mothers used to simulate the birth experience) to “Kenya and Djenne Days.” Kenya represents a country in Africa, while “Djenne” is the name of a city in Mali, Africa. Changed all the characters to Black people, including Black couples, family members, social network, and NICU nurses.
Evidential	Evidential adaptations use epidemiological data reflective of the population.	Examples of evidential adaptations made to the eMB included the following: New evidence on Black maternal health in the United States. New evidence on preterm birth in the United States. New evidence on preterm infants, including how to calculate adjusted age, cognitive development, and things to look out for. New evidence on racial discrimination, obstetric racism, and mental health among Black families. New evidence on stressors in the NICU. New evidence on advocacy strategies in the Black community and the NICU. New materials on pleasant activities that appeal to Black communities and in the NICU. New materials on strategies to balance time as a mom with a preterm infant.
Linguistic	Linguistic adaptations are based on improving the language to suit the culture and context of the audience.	Examples of linguistic adaptations made to the eMB included the following: New language referencing historical ideologies (e.g., having the strength of the ancestors.) New terminologies such as “mama.”
Constituent-involving	Constituent-involving adaptations ensure programs are centered expressly on the experiences of the target group.	Examples of constituent-involving adaptations made to the eMB included the following: Use of a Delphi approach to provide insights into the cultural nuances and idiosyncrasies of Black mothers with PTB to develop the eMB 4 Blackmamas.
Sociocultural	Sociocultural adaptations address health within the broader sociocultural context wherein they exist.	Examples of sociocultural adaptations made to the eMB included the following: New materials containing religious content (e.g., Bible and Quran verses), quotes from spiritual leaders (e.g., Buddha), and poems/musical lyrics from the Black communities encompassing their sociocultural values as strategies to manage mood. New document for journaling.

**Table 3:** Sociodemographic characteristics of experts (*n* = 11).

Variable	Round One N
Mothers	8
Age	8, Range (22–43)
Income levels	
USD 30,000 to USD 49,999	3
USD 70,000 and above	5
Marital status	
Married/living with partner	6
Never married	2
Educational status	
High school or associates	3
Graduate degree or more	5
Previous maternal conditions ^a^	
No	4
Yes	4
Type of birth	
Singleton	8
Type of delivery	
Vaginal birth	2
Emergency cesarean birth	6
Maternal hospitalized after birth	
No	6
Yes	2
Infants living with any health condition	
No	3
Yes ^b^	3
NA ^c^	2
Days spent in the NICU Range (0–109)	-
Gestational week of delivery Range (29–35)	
**Professionals**	3
Age	3, Range (38–63)
Educational status	
Graduate degree or more	3
Sex	
Female	3
Area of specialization	
Healthcare ^d^	2
Advocacy organization	1
Years working in the field	
1–5	2
>15	1

a: gestational diabetes, hypertension, preeclampsia, eclampsia, or other pregnancy-related health condition; b: autism, gastrointestinal issues, eczema, allergies; c: not available; d: virtual clinical and mental health services, therapist.

**Table 4:** Examples of revisions made between Rounds One and Two.

eMB Content	Brief Overview	Round One—Initial Adaptation	Round Two—Enhanced Adaptation Based on Expert Recommendations
Djenne and Kenya Days Vignettes	Additional text was included to acknowledge mothers without immediate family support.	“The thoughts Djenne and Kenya are having are not helpful. This type of thinking, such as “all or nothing thinking,” “doomed future,” “over-generalizations,” and “self-blame,” creates a negative chain of harmful thoughts, which can lead to feeling even worse than before. Kenya calls her mom, who tells her how it is important to notice harmful thoughts. This is how Kenya learns to break the negative chain, have more helpful thoughts, and do more positive things for herself and her baby.”	Includes the text from Round One and the text below: “We acknowledge that support from family or friends may not be readily available for some mothers. Some mothers may also be unwilling to unload their worries onto others. While this can be challenging, we recommend that mothers consider other ways to seek support. For example, mothers can join virtual support groups or channels on Facebook or Instagram for Black families with preterm birth. The resource page lists some activities moms can engage in that may support and influence a positive personal reality. We also created a list of community organizations and centers serving and supporting Black families that can be helpful. As Kenya reached out to her mom, we also encourage mothers to try to reach out even though it may seem that others may not understand their situation.”
	Included additional text to acknowledge communication gaps that can occur between partners in the NICU.	“Djenne and Kenya’s babies have been in the NICU for some time. As they prepare for the baby’s arrival home, they have noticed it is not always easy to have positive interactions with others, especially their partners, who may be having difficulty managing the NICU experience. Sometimes, their partners don’t always feel the most supportive and are focused on things other than helping them. Today, Djenne and Kenya have a NICU visit. Djenne feels worse after having a negative interaction with her partner. She feels defeated and more depressed. Feeling this way, it seems even more difficult to go to the visit at all. Kenya is disappointed in her partner but wonders if there might be a different communication method. By using assertive communication and expressing her needs, she helps her partner understand how important positive social interactions and continuous NICU visitation are for Kenya and her baby.”	Includes the text from Round One and the text below: “As you continue this lesson, you will learn how to identify your support network and communication styles that can help you through this journey. Understanding people in your support networks, particularly those without active or functional support from a partner, can be helpful. We understand that communications may not be as nuanced, but these are some experiences encountered by other Black moms in the NICU.”
	Changed the text of Djenne’s story to provide a more balanced and less extreme perspective.	“I will only care for the baby and her brother when I’m rested.”	“This is really hard; I will not give up. I will push through and take good care of the baby and her brother.”
Additional resources	New materials on how to self-advocate for oneself.	Not included.	Curated a document containing strategies to advocate for self and baby in the NICU based on the literature and anecdotal experiences from Black mothers.

## Data Availability

The data (survey instrument and adapted program) are not publicly available due to privacy and copyright issues. Data may be available on request from the authors.

## Supplementary Materials

The following supporting information can be downloaded at https://www.mdpi.com/article/10.3390/ijerph22081304/s1, Example of Round One and Two questions.

## References

[1] Torres-Ruiz M., Robinson-Ector K., Attinson D., Trotter J., Anise A., Clauser S. (2018). A Portfolio Analysis of Culturally Tailored Trials to Address Health and Healthcare Disparities. Int. J. Environ. Res. Public Health.

[2] Kreuter M.W., Lukwago S.N., Bucholtz D.C., Clark E.M., Sanders-Thompson V. (2003). Achieving Cultural Appropriateness in Health Promotion Programs: Targeted and Tailored Approaches. Health Educ. Behav..

[3] Pew Research Center Facts About the U.S. Black Population | Pew Research Center. https://www.pewresearch.org/social-trends/fact-sheet/facts-about-the-us-black-population/.

[4] Maharjan S., Dhakal L., George L., Shrestha B., Coombe H., Bhatta S., Kristensen S. (2022). Socio-culturally adapted educational videos increase maternal and newborn health knowledge in pregnant women and female community health volunteers in Nepal’s Khotang district. Womens Health.

[5] Valdez C.R., Padilla B., Moore S.M., Magaña S. (2013). Feasibility, Acceptability, and Preliminary Outcomes of the Fortalezas Familiares Intervention for Latino Families Facing Maternal Depression. Fam. Process.

[6] Parker A. (2021). Reframing the narrative: Black maternal mental health and culturally meaningful support for wellness. Infant. Ment. Health J..

[7] Centers for Disease Control and Prevention (2022). Premature Birth Centers for Disease Control and Prevention. https://www.cdc.gov/maternal-infant-health/preterm-birth/index.html.

[8] Frey H.A., Klebanoff M.A. (2016). The epidemiology, etiology, and costs of preterm birth. Seminars in Fetal and Neonatal Medicine.

[9] Carr H., Cnattingius S., Granath F., Ludvigsson J.F., Edstedt Bonamy A.K. (2017). Preterm birth and risk of heart failure up to early adulthood. J. Am. Coll. Cardiol..

[10] Calthorpe L.M., Baer R.J., Chambers B.D., Steurer M.A., Shannon M.T., Oltman S.P., Karvonen K.L., Rogers E.E., Rand L.I., Jelliffe-Pawlowski L.L. (2021). The association between preterm birth and postpartum mental healthcare utilization among California birthing people. Am. J. Obstet. Gynecol. MFM.

[11] Winter L., Colditz P.B., Sanders M.R., Boyd R.N., Pritchard M., Gray P.H., Whittingham K., Forrest K., Leeks R., Webb L. (2018). Depression, posttraumatic stress and relationship distress in parents of very preterm infants. Arch. Womens Ment. Health.

[12] Forcada-Guex M., Borghini A., Pierrehumbert B., Ansermet F., Muller-Nix C. (2011). Prematurity, maternal posttraumatic stress and consequences on the mother–infant relationship. Early Hum. Dev..

[13] Bower K.M., Geller R.J., Perrin N.A., Alhusen J. (2018). Experiences of Racism and Preterm Birth: Findings from a Pregnancy Risk Assessment Monitoring System, 2004 through 2012. Women’s Health Issues.

[14] Giurgescu C., Misra D.P. (2018). Psychosocial factors and preterm birth among black mothers and fathers. MCN Am. J. Matern./Child Nurs..

[15] Larrabee Sonderlund A., Schoenthaler A., Thilsing T. (2021). The association between maternal experiences of interpersonal discrimination and adverse birth outcomes: A systematic review of the evidence. Int. J. Environ. Res. Public Health.

[16] Paradies Y., Ben J., Denson N., Elias A., Priest N., Pieterse A., Gupta A., Kelaher M., Gee G., Hills R.K. (2015). Racism as a Determinant of Health: A Systematic Review and Meta-Analysis. Hills RK, editor. PLoS ONE.

[17] Horbar J.D., Edwards E.M., Greenberg L.T., Profit J., Draper D., Helkey D., Lorch S.A., Lee H.C., Phibbs C.S., Rogowski J. (2019). Racial segregation and inequality in the neonatal intensive care unit for very low-birth-weight and very preterm infants. JAMA Pediatr..

[18] Ajayi K.V., Garney W.R. (2023). What Black mothers with preterm infants want for their mental health care: A qualitative study. Women’s Health Rep..

[19] Ajayi K.V., Garney W.R. (2022). Understanding the Domains of Experiences of Black Mothers with Preterm Infants in the United States: A Systematic Literature Review. J. Racial Ethn. Health Disparities.

[20] Grigoriadis S., VonderPorten E.H., Mamisashvili L., Tomlinson G., Dennis C.L., Koren G., Steiner M., Mousmanis P., Cheung A., Radford K. (2013). The Impact of Maternal Depression During Pregnancy on Perinatal Outcomes: A Systematic Review and Meta-Analysis. J. Clin. Psychiatry.

[21] Mendelson T., Cluxton-Keller F., Vullo G.C., Tandon S.D., Noazin S. (2017). NICU-based Interventions To Reduce Maternal Depressive and Anxiety Symptoms: A Meta-analysis. Pediatrics.

[22] Ponting C., Mahrer N.E., Zelcer H., Dunkel Schetter C., Chavira D.A. (2020). Psychological interventions for depression and anxiety in pregnant Latina and Black women in the United States: A systematic review. Clin. Psychol. Psychother..

[23] Mothers Babies Online Course Home | Mothers Babies Online Course [Internet]. https://www.mothersandbabiesprogram.org/project/mothers-and-babies-online-emb/.

[24] Mothers & Babies Postpartum Depression Intervention & Family Support—Northwestern Mothers & Babies. https://www.mothersandbabiesprogram.org.

[25] Barrera A.Z., Wickham R.E., Muñoz R.F. (2015). Online prevention of postpartum depression for Spanish-and English-speaking pregnant women: A pilot randomized controlled trial. Internet Interv..

[26] Lee E.W., Denison F.C., Hor K., Reynolds R.M. (2016). Web-based interventions for prevention and treatment of perinatal mood disorders: A systematic review. BMC Pregnancy Childbirth.

[27] Le H.N., Perry D.F., Mendelson T., Tandon S.D., Muñoz R.F. (2015). Preventing perinatal depression in high risk women: Moving the mothers and babies course from clinical trials to community implementation. Matern. Child Health J..

[28] Ward E.A., Iron Cloud-Two Dogs E., Gier E.E., Littlefield L., Tandon S.D. (2022). Cultural Adaptation of the Mothers and Babies Intervention for Use in Tribal Communities. Front. Psychiatry.

[29] Tandon S.D., Leis J.A., Mendelson T., Perry D.F., Kemp K. (2014). Six-month outcomes from a randomized controlled trial to prevent perinatal depression in low-income home visiting clients. Matern. Child Health J..

[30] Collins P.H. (2002). Black Feminist Thought: Knowledge, Consciousness, and the Politics of Empowerment.

[31] Jorm A.F. (2015). Using the Delphi expert consensus method in mental health research. Aust. N. Z. J. Psychiatry.

[32] Schmit C., Ajayi K.V., Ferdinand A.O., Giannouchos T., Ilangovan G., Nowell W.B., Kum H.-C. (2020). Communicating with patients about software for enhancing privacy in secondary database research involving record linkage: Delphi study. J. Med. Internet Res..

[33] von der Gracht H.A. (2012). Consensus measurement in Delphi studies: Review and implications for future quality assurance. Technol. Forecast. Soc. Chang..

[34] Okpa A., Buxton M., O’Neill M. (2022). Association Between Provider-Patient Racial Concordance and the Maternal Health Experience During Pregnancy. J. Patient Exp..

[35] Coley S.L., Zapata J.Y., Schwei R.J., Mihalovic G.E., Matabele M.N., Jacobs E.A., Anderson C.K. (2018). More than a “number”: Perspectives of prenatal care quality from mothers of color and providers. Women’s Health Issues.

[36] Onyeaka H., Ajayi K.V., Muoghalu C., Eseaton P.O., Azuike C.O., Anugwom G., Oladunjoye F., Aneni K., Firth J., Torous J. (2022). Access to online patient portals among individuals with depression and anxiety. Psychiatry Res. Commun..

[37] Fang M.L., Canham S.L., Battersby L., Sixsmith J., Wada M., Sixsmith A. (2019). Exploring privilege in the digital divide: Implications for theory, policy, and practice. Gerontologist.

[38] Sieck C.J., Sheon A., Ancker J.S., Castek J., Callahan B., Siefer A. (2021). Digital inclusion as a social determinant of health. NPJ Digit. Med..

[39] National Network of Perinatal Quality Collaboratives (NNPQC) Coordinating Center National Network of Perinatal Quality Collaboratives (NNPQC) Coordinating Center NICHQ—National Institute for Children’s Health Quality. https://www.nichq.org/project/national-network-perinatal-quality-collaboratives.

[40] Medina-Martínez J., Saus-Ortega C., Sánchez-Lorente M.M., Sosa-Palanca E.M., García-Martínez P., Mármol-López M.I. (2021). Health Inequities in LGBT People and Nursing Interventions to Reduce Them: A Systematic Review. Int. J. Environ. Res. Public Health.

[41] Huang Y.T., Ma Y.T., Craig S.L., Wong D.F.K., Forth M.W. (2020). How Intersectional Are Mental Health Interventions for Sexual Minority People? A Systematic Review. LGBT Health.

[42] Ashley W. (2014). The angry black woman: The impact of pejorative stereotypes on psychotherapy with black women. Soc. Work Public Health.

