# Supplementary material for: Cultural Adaptation of the Mothers and Babies Online Course for Black Mothers with Preterm Infants: A Delphi Study
Source: Int J Environ Res Public Health. 2025 Aug 20;22(8):1304. doi: 10.3390/ijerph22081304 (PMC12386181; doi:10.3390/ijerph22081304)
Supplement: Supplementary file 1 [file ijerph-22-01304-s001.zip › ijerph-3681317-supplementary.pdf]

EXAMPLES OF QUESTIONS IN ROUND TWO

This supplementary file only provides two examples of the revisions made based on the expert's feedback, not the edited text or images where the modifications were made.

SECTION 1: DELPHI STUDY- REVISIONS

In Round one, you provided detailed and valuable feedback on the adaptations made to the **eMB for Blackmamas.** Based on your feedback, we have included some items that need final voting.

The old texts are highlighted in **BLUE**.

Updated or included texts are highlighted in **RED.**

LESSON ONE: PURPOSE AND OVERVIEW

In Round one, we introduced the Djenne and Kenya Days. Some respondents mentioned the need to acknowledge the issue of mistrust that affects the healthcare experiences of Black families.

In response, two changes were made.

1. Updated the text introducing the Djenne and Kenya story.
2. Updated the Djenne and Kenya story (the old and new images are included for voting).

Update to Djenne and Kenya story's conversation

We changed Kenya and Lesotho's conversation to reflect the issue of trust.

Q1.1 Does the updated Djenne and Kenya Days address the issue raised?

Yes

No

LESSON TWO: THOUGHTS AND MOOD

In Round one, respondents shared their concerns about the Djenne and Kenya Story, stating that we needed to address the lack of support that some mothers experience.

In response to these concerns, we made the following changes:

1. Updated the text introducing the Djenne and Kenya Days story (we included the image for reference).

Q2.1 Which text do you prefer?

I prefer the text with the "old text" only

I prefer the text with the "old and included text"
